# Dental anxiety is related to postoperative symptoms in third molar surgery

**DOI:** 10.3389/fpsyt.2022.956566

**Published:** 2022-08-18

**Authors:** Feng Qiao, Menghua Zhang, Tingting Zhang, Dongwang Zhu

**Affiliations:** ^1^Department of Oral and Maxillofacial Surgery, School and Hospital of Stomatology, Tianjin Medical University, Tianjin, China; ^2^Graduate School and Hospital of Stomatology, Tianjin Medical University, Tianjin, China

**Keywords:** dental anxiety, postoperative symptom, logistic regression, non–linear relation, lower third molar extraction

## Abstract

**Purpose:**

To examine the association of preoperative dental anxiety with the severity of postoperative symptoms among patients undergoing lower third molar (LM3) extraction surgery.

**Materials and methods:**

We conducted a hospital-based prospective study with a sample size of 213 patients. All the patients underwent LM3 extraction surgery at the Stomatology Hospital of Tianjin Medical University. Preoperative dental anxiety was measured using the Dental Anxiety Scale for Third Molar Surgery (DAS-TMS) and classified into four categories: No anxiety, Some unease, Anxious, and Very anxious. The primary outcome was defined using the postoperative symptom severity scale on the seventh day after surgery. The patients' clinical characteristics, radiologic features, and surgery-related variables were used as control variables. Bivariate analysis involved Fisher's exact test and Kruskal–Wallis test. Multivariable logistic analysis was used to assess preoperative dental anxiety in relation to the severity of postoperative symptoms. We applied a two-piecewise regression model to examine the potential non–linear associations.

**Results:**

The mean (SD) dental anxiety score was 10.56 (3.84). The proportion of dental anxiety was as follows: No anxiety, 7.5%; Some unease, 46.9%; Anxious, 31.0%; Very anxious, 14.6%. The multivariable-adjusted ORs with 95% CIs of postoperative symptoms were 1.00 for No anxiety, 3.63 (0.90–14.68) for Some unease, 5.29 (1.25–22.33) for Anxious, and 4.75 (1.02–22.18) for Very anxious (*P* for trend = 0.047). The risk of serious postoperative symptoms increased with the dental anxiety level up to 7 points (adjusted OR 1.94, 95% CI 1.12–3.74; *P* = 0.012). When the dental anxiety level exceeded 7 points, the level of DAS-TMS was not associated with the risk of serious postoperative symptoms (OR 0.98, 95% CI 0.88–1.08; *P* = 0.756).

**Conclusions:**

Findings suggest that dental anxiety is associated with a risk of serious postoperative symptoms following LM3 removal. The degree of dental anxiety in patients before LM3 extraction surgery should be of concern to clinicians.

## Introduction

Removal of third molars is a common surgical procedure performed in maxillofacial and oral surgery. Patients with wisdom tooth extractions have the highest dental anxiety levels (1, 2). Preoperative dental anxiety results in a delay or avoidance of dental treatment and, consequently, poorer oral health and oral health-related quality of life (3, 4). Recent studies have shown that psychological anxiety leads to the activation of the body's stress reaction and slower postoperative recovery (5). Dental anxiety has also been shown to be associated with postoperative pain (5–7).

Recently, patient recovery has attracted considerable attention in the field of third molar surgery (8–12). Questionnaires on evaluating the severity of postoperative symptoms have become more useful and are widely used (13–15). Additionally, a previous study found that patient anxiety affects the difficulty of impacted lower third molar extraction (16). The surgical difficulty is frequently associated with considerable postoperative adverse effects such as pain, edema, and trismus (9, 17, 18). Previous studies have also indicated that the complexity of the surgical operation has been associated with postoperative symptoms (14). These results suggest that preoperative dental anxiety may be associated with postoperative symptoms. However, the specific relationship between preoperative dental anxiety and the severity of postoperative symptoms remains unclear.

Several studies have evaluated the association between dental anxiety and LM3 surgery (19–21). Patients with high dental anxiety experience greater trismus and more pain (19). Onwuka et al. reported that preoperative dental anxiety is more common in women (21). However, confounding factors have not been fully incorporated into multivariable regression models for control (19). A direct independent association between preoperative dental anxiety and postoperative symptoms in patients undergoing lower third molar removal is still unestablished. Moreover, the significance of the non–linear relationship still requires further clarification. Therefore, exploring the non–linear relationships between anxiety and postoperative symptoms using non–linear methods is important.

The present study assessed the relationship between preoperative dental anxiety and postoperative symptoms after lower third molar (LM3) extraction surgery.

## Methods

### Study design and patients

Between May 2019 and June 2020, we performed a hospital-based prospective cohort study of patients who underwent LM3 extraction surgery at the Department of Oral and Maxillofacial Surgery, Stomatology Hospital of Tianjin Medical University. The inclusion criteria for this study were adult patients who had complete mandibular permanent dentition between 18 and 60 years of age and underwent LM3 extraction surgery under local anesthesia. The exclusion criteria were as follows: individuals aged 17 years or younger, no need for mucosal incision or high-speed turbine for extraction, inability to tolerate the procedure, presence of current pain, edema, trismus, and infection, poor compliance to postoperative care instructions and those who had previously undergone this surgery. Ethical approval was obtained from the Ethics Committee of the Stomatology Hospital of Tianjin Medical University (Tianjin, China) (No: TMUhMEC20210508). This study adhered to the ethical guidelines of the Declaration of Helsinki. Written informed consent was obtained from all patients.

### Evaluation of dental anxiety

Preoperative dental anxiety (exposure) was measured using a self-report questionnaire (Dental Anxiety Scale for Third Molar Surgery, DAS-TMS). DAS-TMS was developed specifically for mandibular third molar extraction and is based on the Chinese version of the Modified Dental Anxiety Scale (22, 23). There were four questions on this scale. Each question was answered by a single choice among five options, representing a score of 1 to 5. The scale had a total score of 4–20. Dental anxiety levels were classified by grouping linear variables on a scale of 4–5 as No anxiety, 6–10 as Some unease, 11–15 as Anxious, and 16–20 as Very anxious. The No anxiety (4–5 points) group was defined as the reference group. We evaluated dental anxiety while sitting in a dental chair, ready for local anesthesia.

The following reliability (*n* = 213) and validity (*n* = 30) of the DAS-TMS were assessed in a randomly selected sample from the study population: (a) internal consistency, (b) temporal stability, and (c) criterion-related validity (i.e., association with the Index of Dental Anxiety and Fear, IDAF-4C) (24). (d) discrimination validity, and (e) the construct validity from a confirmatory factor analysis (CFA).

### Outcome measurement

The total severity of postoperative symptoms was used to gauge the study outcome. Postoperative symptoms were measured with the Postoperative Symptom Severity Scale (13, 15, 25). The full postoperative symptom scale was first proposed by Ruta in 2000, which contains a 7-item subscale: eating, speech, sensation, appearance, pain, sickness, and interference with daily activities (15). The total number of postoperative symptoms was the sum of the subscales. The postoperative symptom score was calculated from the self-reported questionnaire items as follows: Full Postoperative symptom score = eating scores + speech scores + sensation scores + appearance scores + pain scores + sickness scores + interference scores (15). Patients were asked to record postoperative symptoms observed during the first seven days immediately following surgery. Suture removal was done on the seventh day after extraction surgery. For the analysis, patients were classified into two groups (0 = low-risk group and 1 = high-risk group) based on the median total postoperative symptom score (20.89). A higher postoperative symptom score reflects more severe symptoms. The same investigator conducted the follow-up for all patients. Follow-up for the first postoperative week *via* interview was done during suture removal.

### Control of confounders

Standardized tables were used to collect confounding variables for each operation. Demographic variables included sex (male/female) and age. Radiographic variables were specified using Winter's classification (26), the Pell-Gregory ramus classification, and the Pell-Gregory occlusal position (27). The operation time was also included, which was defined as the interval between the first incision and placement of the last suture.

Panoramic films were taken before surgery to evaluate and classify LM3 radiologic variables (Winter classification, Pell-Gregory ramus classification, and Pell-Gregory occlusal position). This classification method is based on the area covered by the leading edge of the mandibular ascending ramus to the teeth (Class I-III) and the depth of impaction relative to the adjacent teeth (Positions A, B, or C) (26–28).

All surgical procedures were performed in the same surgical unit. Local anesthesia was administered with 2% lidocaine and 4% articaine hydrochloride under the same conditions. A full-layer mucoperiosteal flap was elevated, and either of the two techniques was employed: cases that used a triangular flap and cases that did not need a triangular flap. After determining the necessity and extent of bone removal, bone was removed from the occlusal surface of the teeth using a high-speed turbine with sufficient speed and torque. A tungsten steel crack needle drill (NSK Ltd.) was used to section the tooth (29). Machines used during the procedure were obtained from Japan (NSK Ltd., Tokyo, Japan). Patients were given routine medication and wound dressing guidance immediately after surgery. Antibiotics were administered for 3 days after surgery.

### Data management and statistical analysis

All statistical analyses were performed using R version 4.1.0, an open-source language for statistical calculations (R Foundation for Statistical Computing, Vienna, Austria). Continuous variables are expressed as median (interquartile range), while categorical variables are expressed as absolute frequencies [*n* (%)]. Continuous and categorical data were compared using the Kruskal–Wallis test and Fisher's exact test, respectively. Postoperative symptoms (binomial) were used as the dependent variable, and DAS-TMS was used as the independent variable for logistic regression. We built three multivariable logistic models (model 1, model 2, and model 3) to determine the association between preoperative dental anxiety and the severity of postoperative symptoms. Adjustments were not made in the crude model. In model 2, adjustments were made for impaction status (Pell-Gregory ramus classification, Pell-Gregory occlusal position, Winter classification) (categorical variables) and operation time (continuous variables, minutes). In model 3, additional adjustments were made for sex (categorical variables) and age (continuous variables).

We also performed secondary analyses with postoperative symptoms as continuous variables in the multivariable linear regression model. We then explored the relationship between DAS-TMS and postoperative symptoms following LM3 extraction surgery using a smoothing plot with an adjustment for potential confounders. We further applied a two-piecewise regression model to examine the threshold effect of DAS-TMS. A trial method was used to determine the threshold level of DAS-TMS at which the relationship between DAS-TMS and postoperative symptoms began to change and became notable. The trial inflection point was moved along a predefined interval, and the inflection point that gave the maximum model likelihood was detected. Differences were considered statistically significant at a two-sided *P* value of 0.05.

## Results

**Figure 1:**
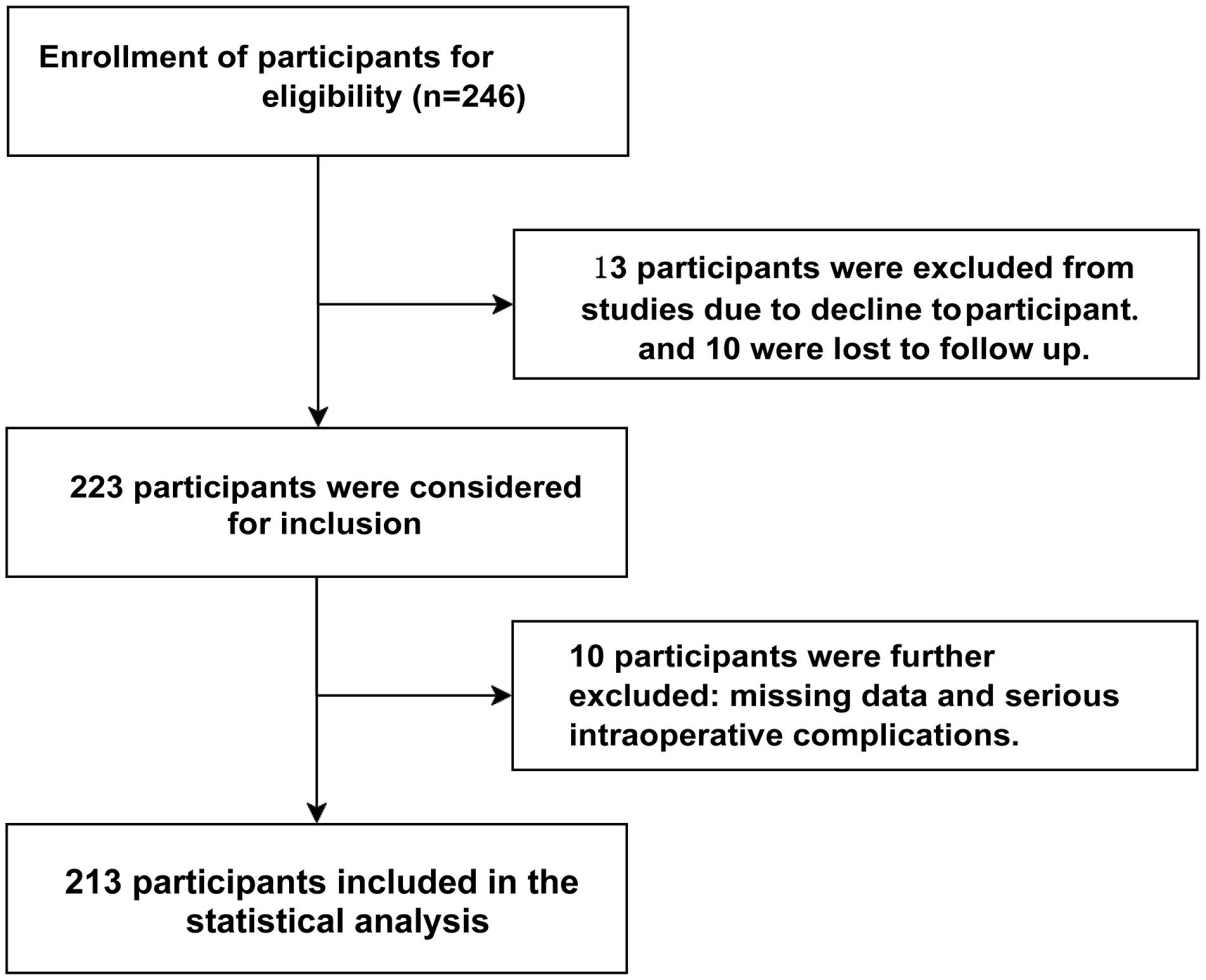
Study participants selection flowchart in final analysis.

A total of 213 patients were enrolled in this study (Figure 1). The baseline patient characteristics are shown in Table 1. The proportion of dental anxiety was as follows: No anxiety, 7.5%; Some unease, 46.9%; Anxious, 31.0%; Very anxious, 14.6%; Overall, the mean (SD) dental anxiety score was 10.56 (3.57), the median (IQR) age was 27.5 (22.0–31.5) years, and 35.7% (76 of 213) were males. The mean (SD) dental anxiety score was 10.22 (3.94) in males and 10.75 (3.34) in females.

**Table 1:** Baseline characteristics of the study population for total samples and subgroups according to categories of DAS-TMS^*^.

**Variables**	***N***	**DAS-TMS**				***P***
		**No anxiety (4–5 points)**	**Some unease (6–10 points)**	**Anxious (11–15 points)**	**Very anxious (16–20 points)**	
Sample size (%)	213	16 (7.5)	100 (46.9)	66 (31.0%)	31(14.6%)	
Postoperative symptoms score > median^#^ No (%)	106	3 (2.8)	48 (45.3)	37 (34.9)	18(17.0)	
Gender, No. (%)						0.019
Male	76	10 (62.5)	36 (36.0)	16 (24.2)	14 (45.2)	
Female	137	6 (37.5)	64 (64.0)	50 (75.8)	17 (54.8)	
Age, median (IQR)		27.5 (22, 31.5)	29 (22, 36)	27 (23,33)	30 (23,45)	0.15
Winter, No. (%)						0.99
Vertical	54	5 (31.3)	24 (24.0)	15 (22.7)	10 (32.3)	
Mesioangular	95	8 (50.0)	45 (45.0)	31 (47.0)	11 (35.5)	
Horizontal	55	3 (18.8)	26 (26.0)	17 (25.8)	9 (29.0)	
Distoangular	3	0 (0.0)	2 (2.0)	1 (1.5)	0 (0.0)	
Inverted	6	0 (0.0)	3 (3.0)	2 (3.0)	1 (3.2)	
PG-ramus, No. (%)						0.3
I	109	8 (50.0)	55 (55.0)	34 (51.5)	12 (38.7)	
II	71	6 (37.5)	33 (33.0)	23 (34.8)	9 (29.0)	
III	33	2 (12.5)	12 (12.0)	9 (13.6)	10 (32.3)	
PG-class, No. (%)						0.76
A	109	7 (43.8)	53 (53.0)	30 (45.5)	19 (61.3)	
B	86	8 (50.0)	38 (38.0)	29 (43.9)	11 (35.5)	
C	18	1 (6.3)	9 (9.0)	7 (10.6)	1 (3.2)	
Operation time, median (IQR)		17.5 (11, 27.5)	20 (14, 29)	20 (15, 30)	25 (20, 30)	0.22

* Continuous variables were verbalized as the median (interquartile range), while categorical variables were verbalized as absolute frequencies, n (%). Continuous and categorical data were compared by using the Kruskal–Wallis test and exact fisher Chi-squared test, respectively.

# Total postoperative symptoms score was classified patients into two groups (0 = low-risk group and 1 = high-risk group) by median (20.89). A higher PoSS score reflected more severe symptoms.

The DAS-TMS revealed good internal consistency (Cronbach's α = 0.905) and temporal stability (ρ = 0.67; *p* < 0.001). The score was significantly correlated with the IDAF-4C score (ρ = 0.63, *p* < 0.001), supporting good criterion-related and discrimination validity. For construct validity, the CFA revealed that the data from the DAS-TMS fit well with the two-factor model (χ^2^ = 0.654, *P* = 0.419, with a root mean square error of approximation = 0, comparative fit index = 1.001, goodness of fit index = 0.998, normed fit index = 0.999).

The prevalence of serious postoperative symptoms across the categories of DAS-TMS scores was 2.8% for 4–5 points, 45.3% for 6–10 points, 34.9% for 11–15 points, and 17.0% for 16–20 points. The distribution of DAS-TMS based on sex was statistically significant (*P* = 0.02). Age, Winter classification, Pell-Gregory occlusal position, Pell-Gregory ramus classification, and operation time were not significantly different across the categories of DAS-TMS (all *P* values > 0.05) (Table 1).

**Figure 2:**
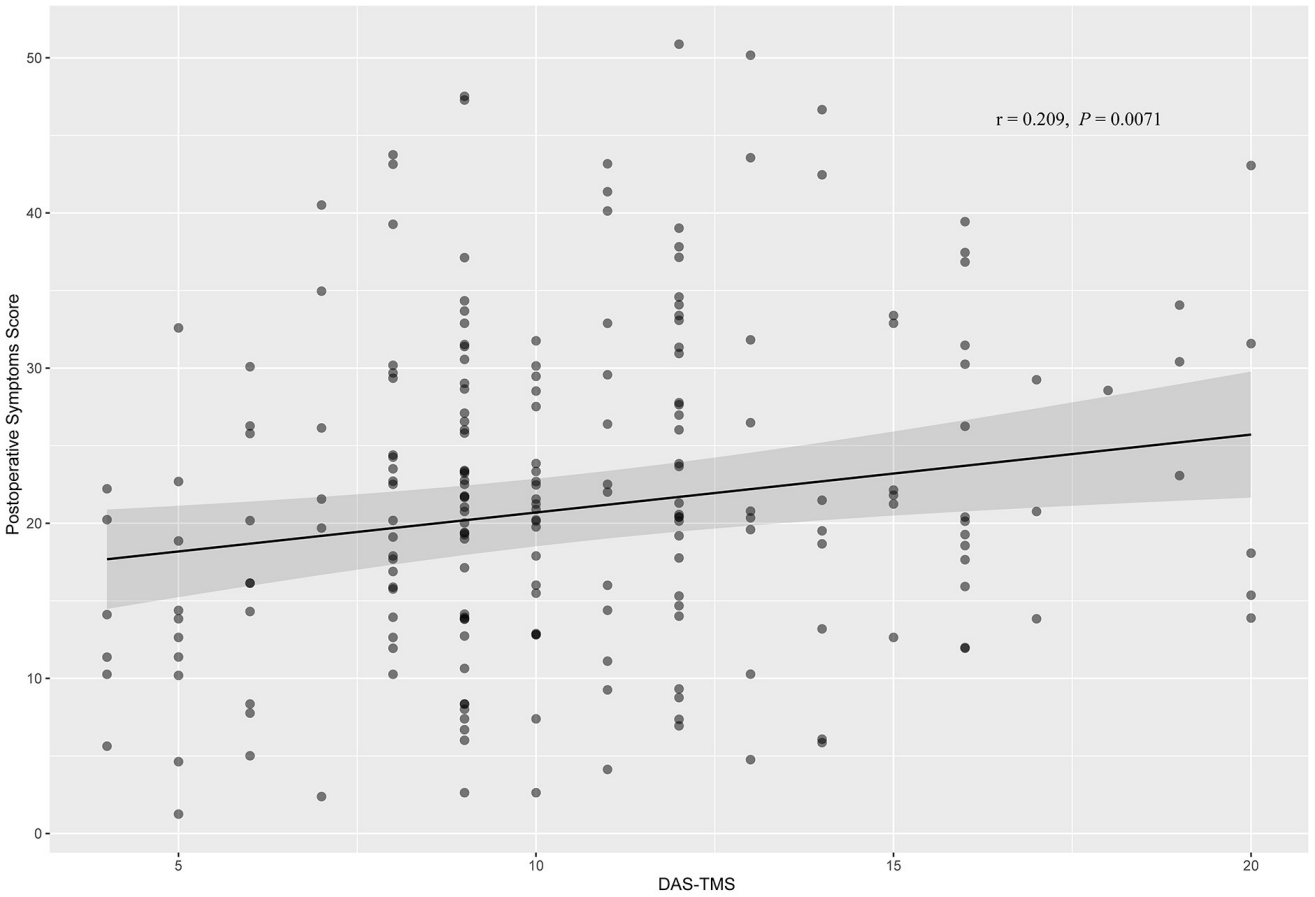
Correlation between DAS-TMS and postoperative symptoms by Pearson's test. DAS-TMS showed slightly positive correlation with postoperative symptoms (*r* = 0.209, *P* = 0.0071).

**Figure 3:**
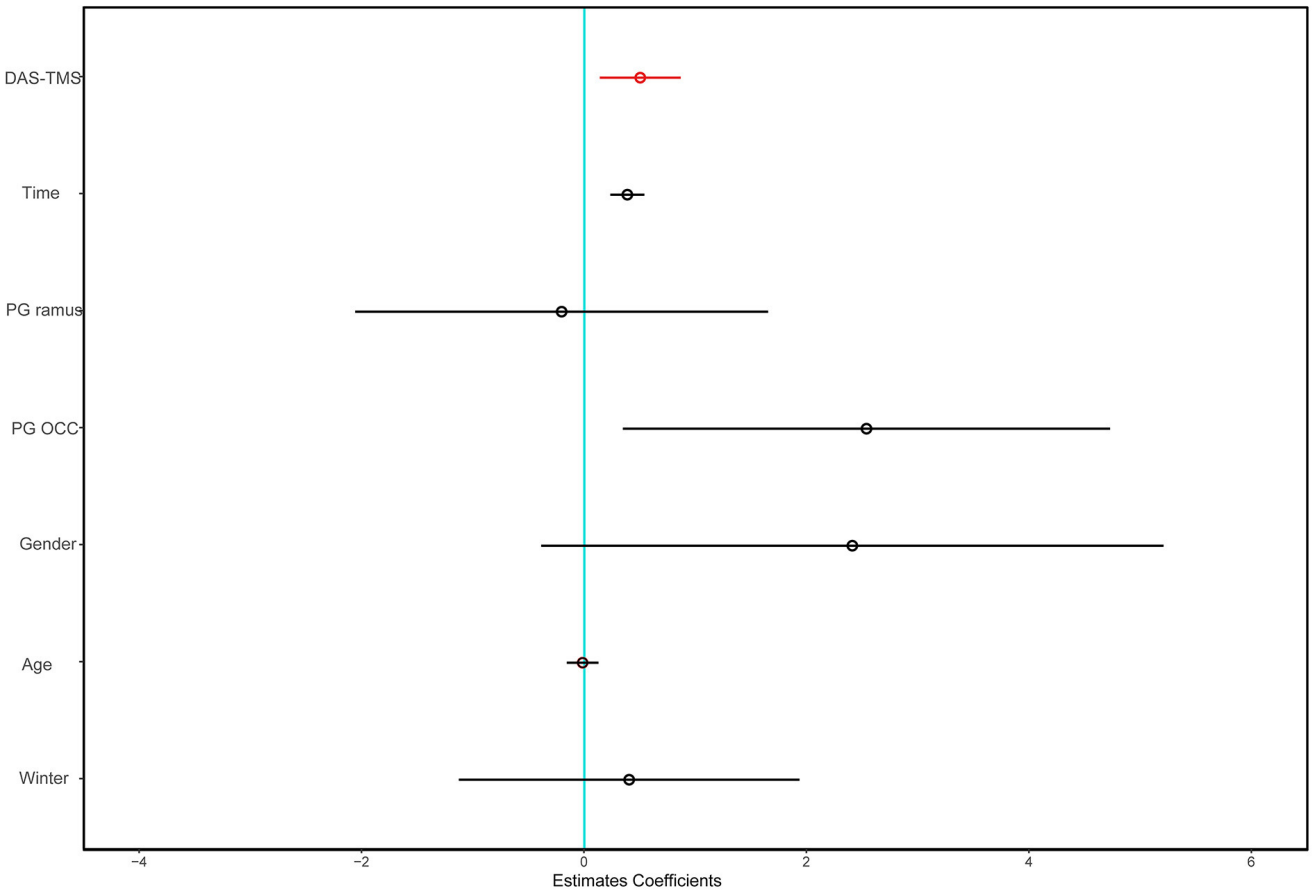
The forest plot for DAS-TMS with postoperative symptoms as a continuous variable.

As shown in Table 2, a higher DAS-TMS level was associated with a higher incidence of postoperative symptoms before multivariate adjustment (Table 2). The multivariate-adjusted ORs (95% CIs) for postoperative symptom severity across categories of DAS-TMS were 1, 3.63 (0.90, 14.68), 5.29 (1.25, 22.33), and 4.75 (1.02, 22.18). The results of the secondary analysis did not significantly change the estimated associations (Figure 2). The forest plot for DAS-TMS with postoperative symptoms as a continuous variable is shown in Figure 3.

**Table 2:** Adjusted association for the categories of the dental anxiety with the severity of postoperative symptom^a^.

**DAS-TMS**		**Model 1^d^**	**Model 2^e^**	**Model 3^f^**
	**N**	**OR (95%CI)**	**OR (95%CI)**	**OR (95%CI)**
4 ~ 5(no anxiety)	16	1.00 (Reference)	1.00 (Reference)	1.00 (Reference)
6 ~ 10(some unease)	100	4.00 (1.07, 14.90)^c^	4.20 (1.06, 16.65)	3.63 (0.90, 14.68)
11 ~ 15(anxious)	66	5.53 (1.44, 21.25)	6.00 (1.46, 24.61)	5.29 (1.25, 22.33)
16 ~ 20(very anxious)	31	6.00 (1.42, 25.42)	5.76 (1.26, 26.33)	4.75 (1.02, 22.18)
*P* for trend^b^	0.019	0.031	0.058

a Multivariate logistic regression analysis was used to sequentially adjusted for covariates.

OR, odds ratio; CI, confidence interval.

b P for trend: P for linear trend was calculated by modeling the median of the dental anxiety for each quintile as a continuous variable.

c Continues variables.

d Crude model.

e Adjusted for impaction status (Pell-Gregory's classification, Pell-Gregory's occlusion, Winter classification) and operation time. Adjusted odds ratios (95% confidence interval) (all such values).

f Additionally adjusted for gender, age.

**Figure 4:**
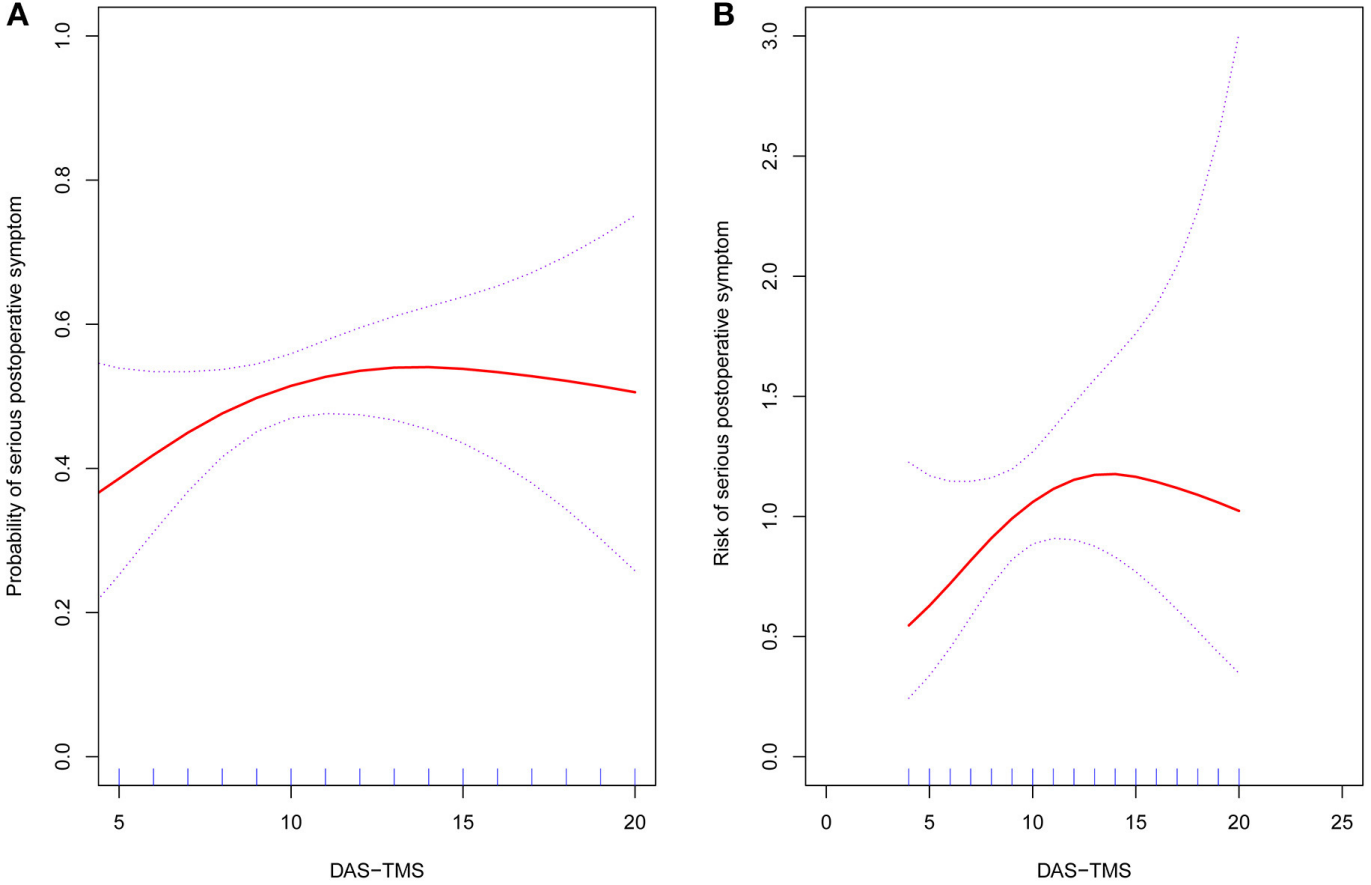
The relationship between DAS-TMS and the risk of severity of postoperative symptoms following LM3 surgery. A non–linear relationship between the DAS-TMS and risk of severity of postoperative symptoms was observed after adjusting for impaction status (Pell-Gregory's classification, Pell-Gregory's occlusion, Winter classification), operation time, gender, and age. **(A)** Probability of serious postoperative symptom; **(B)** Risk of serious postoperative symptom.

After adjusting for these possible factors related to postoperative symptoms, a non–linear relationship between DAS-TMS and postoperative symptoms was observed (Figure 4). The risk of serious postoperative symptoms increased with the dental anxiety level up to 7 points (adjusted OR 1.94, 95% CI 1.12–3.74; *P* = 0.012). When the dental anxiety level exceeded 7 points, the level of DAS-TMS was not associated with the risk of serious postoperative symptoms (OR 0.98, 95% CI 0.88–1.08; *P* = 0.756) (Table 3).

**Table 3:** Threshold effect analysis of DAS-TMS on the severity of postoperative symptoms using piecewise linear regression^a^.

**Inflection point of DAS-TMS**	**Odds ratio**	***P*** **value**
	**(95%CI)**	
≤ 7 point	1.94 (1.12-3.74)	0.012
>7point	0.98 (0.88, 1.08)	0.756

a Adjusted for impaction status (Pell-Gregory's classification, Pell-Gregory's occlusion, Winter classification), operation time, gender, and age.

## Discussion

Dental anxiety is a common problem in patients undergoing third molar extraction (30). Dental anxiety is a significant problem for both patients and dental professionals. Information provided to the patient and dentist concerning dental anxiety is important and based on scientific evidence. Our findings indicate that dental anxiety is associated with postoperative symptoms during third molar extraction surgery. Previous studies indicated that dental anxiety is associated with surgical difficulties and postoperative pain (5, 6, 19). Management of postoperative complications is important for faster recovery. Identifying the relationship between preoperative dental anxiety and associated postoperative symptoms can help minimize and prevent postoperative complications.

Patients with high dental anxiety often require longer operation times (19). Studies have also reported that operation time is related to postoperative analgesia and the severity of postoperative complications (13). The “Very anxious” category of dental anxiety was associated with a high incidence of postoperative symptoms (OR 4.75, 95% CI 1.02–22.18) after multivariate adjustments. This also supports the conclusion that preoperative anxiety is related to postoperative symptoms. The effect of dental anxiety on the risk of serious postoperative symptoms weakened after additional adjustment, indicating that these confounders may be associated with serious postoperative symptoms.

Our results may be due to inflammatory reactions. Preoperative dental anxiety can lead to the change of prostaglandin E2 (PGE2) (31, 32). PGE2 is thought to enhance inflammation by causing vasodilation and increasing the local blood flow (33). An increase in blood PGE2 concentration caused by preoperative anxiety may affect the severity of postoperative complications. Additionally, preoperative anxiety can significantly change the release of Serotonin (5-HT) (34), which is manifested by the increased secretion of 5-HT by platelets, mast cells, and chromaffin cells. Among them, 5-HT3 can directly excite nociceptors or sensitize them through the internal messenger system. 5-HT2A acts on 5-HT2A receptors at the terminals of primary afferent fibers, resulting in aggravation of pain and edema.

In our research center, 54.4% of the patients had a DAS-TMS score of 10 or less. Our research showed that preoperative anxiety is related to the severity of postoperative symptoms. More importantly, the data further show that the risk of severe postoperative symptoms increases with an increase in DAS-TMS levels of up to 7 points. It also shows that early intervention for preoperative dental anxiety is significant in preventing the occurrence of severe postoperative symptoms. Whether the use of preoperative anxiolytic drugs affects postoperative symptoms merits further exploration.

## Limitations

This study has several limitations. First, the sample was selected from only one local hospital. The patients included in this study were relatively young, which may have caused a selection bias. The representativeness of the sample might be limited, and our results may have poor generalizability. Second, the exposure and outcome variables were collected through self–completed questionnaires, which may not reflect the real situation. Third, despite controlling and adjusting many confounders, the existence of residual confounding factors may have affected the results.

## Conclusion

Our findings suggest that preoperative dental anxiety is associated with a risk of serious postoperative symptoms following LM3 extraction surgery. The degree of dental anxiety in patients before LM3 extraction surgery should be of concern to clinicians.

## Data availability statement

The raw data supporting the conclusions of this article will be made available by the authors, without undue reservation.

## Ethics statement

The studies involving human participants were reviewed and approved by Ethics Committee of Stomatology Hospital of Tianjin Medical University (Tianjin, China) (No: TMUhMEC20210508). The patients/participants provided their written informed consent to participate in this study.

## Author contributions

FQ and DZ contributed to the study concept and design. Material preparation, data collection and analysis was performed by FQ, MZ, and TZ. The first draft of the manuscript was written by FQ. All authors contributed to the article, commented on previous versions of the manuscript and approved the submitted version.

## Funding

This work was supported by the Science and Technology Project of Tianjin Health Committee (Grant Number: ZC20134) and the Joint Research Fund of Tianjin Medical University-DeepWise Health care (Grant Number: 1KQ026).

## Conflict of interest

The authors declare that the research was conducted in the absence of any commercial or financial relationships that could be construed as a potential conflict of interest.

## Publisher's note

All claims expressed in this article are solely those of the authors and do not necessarily represent those of their affiliated organizations, or those of the publisher, the editors and the reviewers. Any product that may be evaluated in this article, or claim that may be made by its manufacturer, is not guaranteed or endorsed by the publisher.
